# Risk factors for common mental disorders in health care workers in the city of Diamantina, state of Minas Gerais

**DOI:** 10.47626/1679-4435-2020-574

**Published:** 2021-02-11

**Authors:** Igor Lucas Geraldo Izalino de Almeida, Ana Carolina Monteiro Duarte, Mariana Lopes Simões, Danielle Sandra da Silva de Azevedo, Marcus Alessandro de Alcantara

**Affiliations:** 1 Departamento de Fisioterapia, Universidade Federal dos Vales do Jequitinhonha e Mucuri (UFVJM) - Diamantina (MG), Brazil; 2 Programa de Pós-Graduação em Reabilitação e Desempenho Funcional, Departamento de Fisioterapia, UFVJM - Diamantina (MG), Brazil; 3 Departamento de Enfermagem, UFVJM - Diamantina (MG), Brazil

**Keywords:** health care workers, common mental disorders, primary care

## Abstract

**Introduction:**

The increased prevalence of mental health symptoms in primary care workers in recent years is a major cause of concern, and highlights the need to identify modifiable risk factors for mental health disorders in this population.

**Objectives:**

To analyze the prevalence of common mental disorders and associated factors in primary care workers in the city of Diamantina, state of Minas Gerais.

**Methods:**

This was a cross-sectional, exploratory study involving 203 workers in different sectors of primary health care. Participants completed the Self-Report Questionnaire (20 item version), the Job Stress Scale (JSS), and a form with questions regarding sociodemographic factors, occupational characteristics, lifestyle and habits.

**Results:**

The prevalence of common mental disorders in the sample was 20.2%. These disorders were significantly associated (p ≤ 0.05) with younger age, poor self-assessed health, poor sleep quality and physically demanding work.

**Conclusions:**

Poor working conditions may directly and indirectly affect the occurrence of mental health disorders by influencing lifestyle and habits. The restructuring of organizational norms, together with worker support policies, may positively contribute to the mental health of workers.

## INTRODUCTION

Common mental disorders (CMDs) are conditions characterized by psychogenic symptoms consistent with undiagnosed depression or anxiety. In addition to symptoms such as fatigue, insomnia, forgetfulness, irritability and difficulty concentrating, CMDs may cause functional impairment and psychological distress.^^1^,^2^^

The 2010 Global Burden of Disease Study, which analyzed causes of morbidity and mortality in 85 countries across 19 regions of the world, found that mental illness was the main cause of disability and non-fatal disease burden in the past few decades, as well as a major cause of years lost to premature mortality and years lived with disability.^^3^^

The health of health care workers has received increasing attention in recent years, and the risk factors to which these professionals are exposed must be better understood.^^4^^ Studies have noted that that the proportion of dissatisfied workers across occupations has recently increased.^^5^^ This is attributable to the loss of professional autonomy, pressure from superiors and clients, lack of professional recognition, poor working conditions and low salaries.^^6^^

The evolution of work processes in primary health care due to recent reforms has led to an increase in the responsibilities of health care workers, who are expected to be in closer contact with patient families as they strive to provide more humanized care and address problems at a local level.^^2^^ However, health care workers often have limited access to necessary resources, including basic supplies, autonomy, management support or assistance from colleagues to care for their patients.^^7^^

The difference between what is expected and what is offered to the population in terms of health care has a direct impact on health care workers, resulting in physical and mental exhaustion, loneliness and work-related vulnerability.^^8^^ Recent studies have identified a significant increase in the prevalence of CMDs in health care workers, with these issues overtaking musculoskeletal problems as the leading cause of absenteeism and work-related disability in most developed countries.^^3^^

A multicenter study of primary care workers in southern and northeastern Brazil found a mean prevalence rate of 16% for CMDs.^^2^^ This figure was lower than that observed in specific health sectors, such as community health workers,^^9^^ mental health professionals^^4^^ and nurses.^^10^^

Despite the variability in prevalence rates, CMDs are a common occurrence in this population, affecting 1 in 5 health care workers on average. These findings emphasize the need for monitoring and interventions to improve the mental health of these individuals. The focus on specific populations limits cross study comparisons,^^5^^ and points to the need for further investigation of primary health care workers in different settings.

The need for studies of primary care workers is emphasized in Protocol No. 008/2011 of the Permanent Negotiation Group of the Unified Health System (Sistema Únicode Saúde; SUS), the department responsible for the National Policy Guidelines on Health Promotion for SUS Workers (Diretrizes da Política Nacional de Promoção da Saúde do Trabalhador do SUS).^^11^^ The aim of this policy was to improve the health status of SUS workers through general health surveillance activities. In order to plan and implement preventive measures before the onset of permanent symptoms, it is crucial to understand the characteristics of different health care sectors and their effects on workers’ mental health.

Since primary care is the main point of entry into the health care system, it is important to understand the needs of workers in this sector. The aim of this study was therefore to analyze the prevalence of CMDs and their association with individual and occupational risk factors in a sample of primary care workers in the municipal health system of the city of Diamantina, in the state of Minas Gerais, Brazil.

## METHOD

### STUDY DESIGN

This was an observational, cross-sectional study based on a survey of health and working conditions completed by employees of the Municipal Health Department of the city of Diamantina, in the state of Minas Gerais.

### PARTICIPANTS

The target population consisted of workers employed in seven primary care units, two psychosocial care centers (Centros de Atenção Psicossocial; CAPS), a polyclinic, a pharmacy, a laboratory, a warehouse, a transportation department and a central management unit. The sample also included workers from the health, environmental and epidemiological surveillance sectors.

At the time of the study, the target population consisted of 374 individuals. After excluding workers in rural areas (n = 55), those contracted to other institutions (n = 15) and those on medical leave or vacation (n = 47), 257 workers were eligible for inclusion in the study. A total of 203 workers agreed to participate in the study (response rate = 79%).

Prior to data collection, all interviewers received an instruction manual written by the research coordinators, containing information about the study and answers to possible questions. The research team also took part in a training program to ensure consistent language and procedures were used by all interviewers. Interviews were conducted in private settings, with only the researcher/interviewer present. The project was approved by the Research Ethics Committee of the Universidade Federal dos Vales do Jequitinhonha e Mucuri under project number 1.739.249 (CAAE: 56754616.3.0000.5108), and all participants provided written consent before entering the study.

### VARIABLES STUDIED

The dependent variable was assessed using the Self-Reporting Questionnaire (SRQ-20) adapted to Portuguese, which has been shown to have adequate psychometric properties.^^12^^ The instrument contains 20 questions which screen for mental health symptoms such as depression, anxiety, insomnia, irritability, fatigue, difficulty concentrating, forgetfulness and somatic complaints. The cut-off point for CMDs was defined as 6 or more positive responses for men and 8 or more positive responses for women.^^12^^

Explanatory variables were grouped into three clusters: 1) sociodemographic data (gender, age, education and personal income); 2) health status (self-assessed health, leisure activities and sleep quality); and 3) working conditions (physical demands, physical environment, occupational stress and social support at work). A structured form was used to collect data on gender (male/female), age (years), education (primary/secondary/higher) and personal income (minimum wages). Participants were later classified according to age quartiles.

Self-assessed health was measured using the following question: “How would you rate your general health status?” This question was answered on a 5-point Likert scale. Responses were later dichotomized into positive (good to excellent) and negative (fair to very poor) categories.

Leisure activities were assessed using the following question: “Do you practice leisure activities?” Possible answers were “Yes” or “No.” Sleep quality in the previous month was assessed using the following question: “How would you rate your sleep quality overall for the past month?” This question was answered on a 4-point Likert scale ranging from excellent to very poor.

Occupational stress was evaluated using the Job Stress Scale (JSS), whose psychometric properties have been found to be adequate.^^13^^ The scale examines the following domains: psychological demands, control and social support at work. The psychological demands domain examines time pressure, concentration levels, task interruption and the need to wait for other colleagues to finish their tasks. The control domain evaluates the use and development of decision-making authority and ability. Median scores on these dimensions were used to divide patients according to low- and high-demand, as well as low- and high-control.^^13^^ The demand and control dimensions were then used to classify participants into one of four quadrants: high demands/low control (high-strain), which suggests exposure to high levels of occupational stress; high demands/high control (active work), the most positive combination of the two dimensions; low demands/ low control (passive work), which suggests intermediate exposure to occupational stress; low demands/high control (low-strain), corresponding to the absence of occupational stress. Social support at work was assessed separately. This domain pertains to relationships with colleagues and superiors. Median scores on this subscale were also used to classify participants as having high vs low social support at work.^^13^^

Physical demands were evaluated using six questions regarding participants’ opinions on the physical aspects of their job. The questions examined the frequency of inadequate postures, occupational standing and sitting, excessive movement, weight bearing and lack of regular breaks. These questions were answered using the following scale: 0 = never, 1 = rarely, 2 = sometimes and 3 = always. Higher scores corresponded to a heavier workload. The sum of scores for these items was used as a measure of physical demand. A median split was performed to divide participants into low vs high demand groups.

The quality of the physical environment was evaluated using six questions on ventilation, temperature, lighting and furniture, all answered on a 5-point Likert scale ranging from excellent to very poor. Participants were also asked to rate the internal and external noise levels to which they were exposed at work on a scale ranging from negligible to unbearable. The physical environment variable was operationalized as the sum of the aforementioned items. A median split was also used to categorize each individual’s work environment as adequate (values equal to or below the median) or inadequate (values above the median).

### DATA ANALYSIS

The analyses were conducted using the STATA 12.0 software. After descriptive methods were used to evaluate variable distributions, continuous and discrete variables were categorized for subsequent analysis. The association between explanatory variables and CMDs was assessed using bivariate analyses to calculate prevalence ratios and confidence intervals. All variables significantly associated with CMDs at p < 0.20 were entered into a **multivariate** Poisson regression model with robust variance estimation.

Variables were entered into the multivariate model in three steps: sociodemographic data, health status and, lastly, working conditions. The effects of variables in later steps were therefore adjusted for those of variables in earlier steps, allowing for the influence of each factor to be interpreted relative to previously entered data.^^14^^ Variables with no significant association to CMDs were excluded prior to the next step in the regression, so that the final model contained only the variables whose association with the dependent variable remained significant at p < 0.05.

Multicollinearity in the final model was tested using the variance inflation factor (VIF). Multicollinearity was considered when the condition index value was over 30, when a component explained at least 90% of the variance of two or more variables, if tolerance was below 0.1 or the VIF value was greater than 10.^^15^^

## RESULTS

The final sample consisted of 203 participants, most of whom were community health workers (n = 46; 22.7%), endemic disease control agents (n = 27, 13.3%), nursing technicians (n = 23; 11.3%), nurses (n = 13; 6.4%) and janitorial staff (n = 13; 6.4%). The sample also included physicians, dentists, pharmacists, physical therapists, general service workers, transportation personnel, among other occupational categories. Most participants worked in primary care units (n = 95; 46.8%), a zoonosis management center (n = 31; 15.3%) or the polyclinic (n = 16; 7.9%). The prevalence of CMDs and the distribution of variables in the sample are shown in Table 1.

**Table 1:** Number of observations, frequency and prevalence of common mental disorders (CMDs) according to the characteristics of employees of the Unified Health System (Sistema Único de Saúde; SUS) in Diamantina, state of Minas Gerais, 2017.

Variables	Sample characteristics	
	Total n (%)	Prevalence of CMDs n (%)
Individual characteristics		
Gender		
Male	59 (29.10)	5 (8.47)
Female	144 (70.90)	36 (25.00)
Age group (years)		
20-23	55 (27.00)	14 (25.45)
24-36	47 (23.20)	10 (21.28)
37-60	54 (26.60)	11 (20.37)
> 60	47 (23.20)	6 (12.77)
Education		
Primary	113 (55.70)	25 (22.12)
Secondary or higher	90 (44.30)	16 (17.78)
Personal income (minimum wages)		
1-3	166 (81.80)	39 (23.49)
3 or more	37 (18.20)	2 (5.41)
Health status		
Self-assessment		
Good/excellent	145 (71.40)	17 (11.72)
Fair or lower	58 (28.60)	24 (41.38)
Leisure activities		
Yes	138 (67.90)	28 (20.29)
No	65 (32.10)	13 (20.00)
Sleep quality		
Excellent	64 (31.50)	4 (6.25)
Good	83 (40.90)	12 (14.46)
Poor	36 (17.70)	17 (47.22)
Very poor	20 (9.90)	8 (40.00)
Working conditions		
Physical demands (overload)		
Low	109 (53.70)	13 (11.93)
High	94 (46.30)	28 (29.79)
Physical environment		
Adequate	89 (43.90)	13 (14.61)
Inadequate	81 (39.90)	22 (27.16)
Occupational stress		
Active work	38 (18.80)	8 (21.05)
Low strain	44 (21.60)	6 (13.64)
Passive work	67 (33.00)	13 (19.40)
High-strain	54 (26.60)	14 (25.93)
Social support at work		
Low	92 (45.30)	23 (25.00)
High	111 (54.70)	18 (16.22)

The overall prevalence of CMDs was 20.2%. Women in younger age groups with lower education levels and income had the highest prevalence of CMDs.

Most participants were female, with a mean age of 38.9 (SD, 10.7) years. The largest age group in the sample was that of 20-to 23-year-olds. Most workers had only completed primary education, and earned three minimum wages or less.

The analysis of health status revealed that 28.6% of participants rated their health as less than good. Nearly 32% did not practice leisure activities and 27.6% reported poor to very poor sleep quality in the previous month.

Measures of physical working conditions showed that approximately half the sample experienced high physical demands at work. Additionally, 39.9% described their work environment as inadequate.

A significant proportion of workers were classified in the passive work and high-strain categories, and a similarly high number of workers had low social support at work.

Table 2 shows the results of bivariate analyses of the prevalence of CMDs in health care workers. The following variables were associated with CMDs at p < 0.20: gender, age group, personal income, self-assessed health, sleep quality, social support at work, physical demands and physical work environment.

**Table 2:** Bivariate analysis of the prevalence of common mental disorders (CMDs) according to the characteristics of employees of the Unified Health System (Sistema Único de Saúde; SUS) in Diamantina, state of Minas Gerais, 2017.

Variables	CMDs	
	PR	95%CI
Individual characteristics		
Gender		
Male	1.00	
Female	2.95	1.21-7.16
Age group (years)		
20-23	1.00	
24-36	0.83	0.40-1.70
36-60	0.80	0.39-1.60
> 60	0.50	0.20-1.20
Education		
Primary	1.00	
Secondary or higher	0.80	0.45-1.41
Personal income (minimum wages)		
1 to 3	1.00	
3 or more	0.23	0.57-0.91.
Health status		
Self-assessment		
Good/excellent	1.00	
Less than good	3.52	2.05-6.07
Leisure activities		
Yes	1.00	
No	0.98	0.54-1.77
Sleep quality in the past month		
Excellentgood	1.00	
Poor/very poor	1.92	1.51-2.43.
Working conditions		
Physical demand (overload)		
Low	1.00	
High	2.49	1.37-4.54.
Physical environment		
Adequate	1.00	
Inadequate	1.85	1.00-3.44
Occupational stress		
Active work	1.00	
Low strain	0.64	0.24-1.70
Passive work	0.92	0.41-2.02
High-strain	1.23	0.57-2.64
Social support at work		
Low	1.00	
High	0.64	0.37-1.12.

Significant at 20%. 95%CI: 95% confidence interval; PR: prevalence ratio.

The variables significantly associated with CMDs (p < 0.20) in the bivariate analysis were then included in the multivariate analysis (Table 3). The Poisson regression analysis with robust variance showed that age group was the only individual characteristic to remain significant in the final model. In the second block of variables, only self-assessed health and sleep quality in the past month remained significantly associated with CMDs (p ≤ 0.05). Physical demand was the only work-related variable to remain in the model. No multicollinearity was identified between independent variables.

**Table 3:** Final model for the prevalence of common mental disorders (CMDs) according to the characteristics of employees of the Unified Health System (Sistema Único de Saúde; SUS) in Diamantina, state of Minas Gerais, 2017.

Variables	CMDs Adjusted model	
	PR	95%CI
Age group (years)		
20-23	1.00	
24-36	0.97	1.53-1.82
36-60	0.70	0.40-1.21
> 60	0.37	0.15-0.88
Self-assessed health		
Good/excellent	1.00	
Less than good	3.03	1.74-5.26.
Sleep quality		
Excellent/good	1.00	
Poor/very poor	1.64	1.24-2.16.
Physical demands (overload)		
Low	1.00	
High	2.41	1.44-4.03.

95%CI: 95% confidence interval; PR: prevalence ratio.

## DISCUSSION

This study investigated the association between CMDs, individual characteristics and occupational variables in primary care professionals in Diamantina, state of Minas Gerais. The findings revealed that CMDs have multifactorial causes, and interventions should address the complexity of these conditions.

The prevalence of CMDs identified in this study was higher than that noted in a study of primary care workers in southern and northeastern Brazil,^^2^^ but lower than that observed in health care professionals, community health workers^^4^^ and primary care personnel in the city of Botucatu, in São Paulo.^^16^^ However, the frequency of CMDs was also higher than observed in primary care professionals in Feira de Santana,^^17^^ state of Bahia. The differences between these findings may be at least partly explained by methodological differences in the operationalization of CMDs and the lifestyle or occupational variables investigated.

Despite the variability in previous findings, the results of the present study are consistent with both the national^^18^^ and international literature^^19^^ which identify CMDs as a major concern among health care workers. CMDs can affect both the workers themselves and their activities as health care providers,^^20^^ which in turn can have several individual, economic and social repercussions.

The prevalence of CMDs was lower in older age groups. These findings contrast with those of previous studies suggesting that the prevalence of CMDs in the general population tends to increase with age,^^21^^ but is in line with the higher prevalence of CMDs identified among young doctors in the city of Salvador,^^22^^ in the state of Bahia. The experience acquired over the course of a long career may increase the control and use of skills to carry out different activities, and contribute to the recognition of the social role of workers.^^21^^ The increase in resilience and professional competencies may confer a degree of protection against associated with greater protection against mental health problems.^^23^^ Yet the present findings may also have been influenced by the “healthy worker effect” since those exposed to poor working conditions, such as excessive job demands and occupational stressors, may have already developed physical or mental illnesses which have led them to leave their jobs.^( 24)^

The association between self-assessed health and CMDs observed in this study is also consistent with previous findings. Self-assessed health is a reliable indicator of objective and subjective well-being, as well as a significant predictor of morbidity and mortality,^^25^^ which is why this variable is widely used in epidemiological studies. Recent studies have also shown that divergent work activities, excessive workloads and dissatisfaction with quality of life may contribute to negative perceptions of health among health care workers.^^26^^

Sleep quality was another risk factor which influenced the occurrence of CMDs in the present study. Sleep deprivation is a problematic issue, as it affects memory and attention, and can cause physical and emotional symptoms.^^27^^ These issues are often associated with night work, which is known to affect biological equilibrium, eating habits, the accuracy of job performance, as well as family and social life.^^28^^ Since participants in the present study were not exposed to night work, further research is required to better understand the impact of poor sleep quality on primary care health professionals.

High physical demands were also associated with CMDs in this sample. This was an expected finding, since previous studies have already observed an increased prevalence of CMDs in workers exposed to high physical demands.^^12^^ High workloads compromise workers’ physical and mental health, interfering with workplace relationships and productivity.^^29^^

Unexpectedly, the dimensions of occupational stress were not associated with the occurrence of CMDs. The negative effects of high psychological demands and low social support on health care workers have been discussed in previous studies.^^29^,^30^^ Given the particular characteristics of health care work, such as constant teamwork and the need to establish interpersonal relationships, it seems imperative that services and tasks be designed in a way that supports health care workers without placing them under excessive demands.

With regards to the strengths and weaknesses of this study, it is important to mention that its cross-sectional design may limit the establishment of causal relationships between variables. Comparisons with other populations should therefore be conducted with care. Nevertheless, the convergence between the present findings and the scientific literature supports the external validity of our conclusions.

## CONCLUSIONS

This study found that the prevalence of CMDs was higher among younger workers, with poorer selfassessed health, poor sleep quality and physically demanding jobs. The association of CMDs to individual and occupational variables suggests that this is a complex phenomenon which should be continually monitored in the workplace. While the population of primary care patients grows exponentially every month, the number of health care workers does not increase at the same rate, leading to overburdened workers, who are therefore at higher risk for CMDs. Those in management positions should consider reorganizing tasks and adopting a more democratic management style including worker protection policies and the creation of dedicated spaces for collective problemsolving. These measures may be especially relevant in the case of primary care workers, whose actions are tied to the very efficiency of the Unified Health System.
